# The value evaluation model design and business innovation for P-RAN based on multi-party interaction

**DOI:** 10.1038/s41598-023-30602-2

**Published:** 2023-03-13

**Authors:** Fanrong Meng, Hua Liu, Weimin Li, Xin Chen

**Affiliations:** China Telecom Research Institute, Beijing, 102209 China

**Keywords:** Computational science, Computer science, Information technology

## Abstract

This paper takes the P-RAN, a proximity wireless access network system for 5G and 6G, as an example, and uses the theory related to value evaluation to discuss how to realize the decentralization of underlying infrastructure from the perspective of different interactive players in the system. The analysis of P-RAN helps to promote mobile network operators to recognize network interaction and solve the problems of weak coverage, reduce the cost of network construction, and provides a new research idea for exploring more network evaluation indicators in the future.

## Introduction

With the advent of Web3.0 era, many new applications based on VR/AR and block chain technology are born. The birth of these new applications is inseparable from the support of network systems with large bandwidth, high speed and low latency. Furthermore, with the continuous iterative development of mobile communication technology, the network is bound to develop to a higher frequency. However, there are many problems in high-frequency wide coverage, such as high construction cost, serious interference, large transmission loss and so on, which make it urgent to launch a new network system to support the new business. Qi Bi, chief expert of China Telecom (2022)^1^ in order to solve the loss problem of indoor network through wall, proposed Proximity Radio Access Network(P-RAN), a wireless access network for 5G and 6G. P-RAN is composed of proximity link (PL), cellular link (CL) and other possible non terrestrial link (NTL) (such as satellite link), which uses idle intelligent terminals through Wi-Fi D2D (Device-to-Device) to form a large network where different interactive players obtain different values under the D2D connection, ultimately realizing the self-evolution of the whole network system, It is an important conjecture for exploring decentralized distributed network infrastructure. Relying on the inherent flexibility of mobile network operator (MNO), P-RAN offloads traffic from the core network through Wi-Fi D2D, which is a more flexible and lower cost networking method than base station communication. This means that MNO has taken a real step in reducing the energy consumption per bit, realizing low-cost networking, and even supporting proximity services such as social networking^2–4^. Due to these technical characteristics of D2D, P-RAN has two main application scenarios for the parties interacting. Firstly, in areas with dense terminal equipment and high traffic load, P-RAN can relieve the traffic pressure of base stations and improve users’ quality of experience (QoE). Secondly, in indoor weak coverage or suburban weak coverage or no coverage scenarios, P-RAN can connect to the terminal relay with network coverage through single-hop or multi-hop transmission, and then connect to the cellular network, helping MNO to realize ubiquitous network connections.

At present, there is no specific product launch of P-RAN. However, academic research on technical solutions related to D2D, the key technology of P-RAN, is relatively mature and mainly focuses on the following three areas. First, resource allocation^5–7^; based on the coalitional game, different algorithms and optimization frameworks are proposed for the construction of D2D social networks and D2D cellular networks, so as to solve the resource allocation problem of D2D communication. Second, security and privacy^8–10^; the corresponding solutions are proposed for the medical industry, wireless network transmission cases, and social network cases with high data sensitivity security requirements, thus solving the security privacy problem of D2D transmission. Third, energy efficiency^11–13^; many approaches have been proposed for energy saving in IoT networks, interference immunity in D2D cellular networks, and energy efficiency optimization of D2D heterogeneous network devices. Compared with D2D-related research, the research direction of P-RAN mainly focuses on the implementation of solutions. For example, Zhang et al.^14^ proposed a solution for dynamic multi-hop networking and cellular data offloading, which proves that P-RAN can improve the performance of cell edge through its mobility and flexibility, and P-RAN is considered by MNO as an enabler of 6G offloading strategies. Lu et al.^15^ explored the energy consumption measurement of P-RAN and proved that P-RAN is justified.

In a comprehensive view, besides technical solutions, there is still room for further exploration of P-RAN business model and on-site implementation. Therefore, there is practical implications to explore 6G business innovation by using P-RAN as an example. In the next part of this paper, the relationship between value proposition and business innovation will be elaborated in the literature review, and the value proposition evaluation model will be designed based on this theory.

## Literature review

### Value proposition

Lawrence Miles (1994)^16^ said that value means a great many things to great many people because the term value is used in a variety of ways.

*Value is often confused with cost and with price. The value to producer is different from that to user. Furthermore, the same item may have different values to customers depending on time, place and use.*

Kambil et al.^17^ believed that the common definition of value relies on the price quality ratio of a product or the difference between perceived benefits (the extent to which a product or service fulfills or exceeds costumers needs) and perceived costs (the full costs associated with the product or service).

The term of value proposition was firstly proposed by Lanning and Michaels^18^ from an action perspective, specifically:

*Analyzing customer segments by attributes that customers consider valuable; assessing opportunities in each segment to deliver superior value; and explicitly selecting value propositions that optimize those opportunities.*

And later, Lusch et al.^19^ defined the value proposition from the field of marketing management as:

*The promises a seller makes to his/her customers in terms of value-in-exchange and value-inuse.*

Payne et al.^20^ defined the value proposition from a marketer’s perspective and highlighted that (1) value proposition's critical role as a communication device, (2) the role of resources and resource sharing, and (3) the need for an appropriate package of value that is differentiated from and superior to competitive offerings. Taylor et al.^21^ pointed out that interaction is the fundamental unit of analysis to conceptualize value proposition.

According to the above definition, the value proposition is to analyze the customer's value change from the seller's perspective and interaction is the key point to analyze the value. Scholars often talk about value propositions in terms of stakeholders^22–24^, such as corporate value propositions^25^, and customer value propositions^20,26^. This article will focus on the MNO value proposition as well as the customer value proposition in an integrated manner.

### Value proposition and business innovation

Business innovation represents an often underutilized source of future value, and the presence of each value driver enhances the value creation potential of the business model^27^. Brandenburger and Stuart Jr^28^ believed that the business model is about creating total value for all parties involved. Zott and Amit^29^ pointed out that business models create value for all the parties involved (the focal firm, its partners, suppliers and customers), that is, value co-production.

Frow and Payne^22^ believed that value propositions can play an important role in identifying value co-production in business innovation and creating a stable relationship in stakeholder relations. According to Afuah and Tucci^30^, business model refers to “the method by which a firm builds and uses its resources to offer its customers better value and to make money in doing so”. Johnson et al.^31^ pointed out that business innovation involves the firm’s value proposition.

Most studies have shown that one of the important elements of a business model is the value proposition^32^. In the future 6G era, decentralization will become the norm and the subjects of business model value co-production will be more equal. Based on the P-RAN distributed solution^1^, this paper analyzes the business of P-RAN by specifying MNO and customers’ value proposition to study how to realize the business innovation of value co-production theory in the decentralization era.

### Value proposition evaluation indicators

**Figure 1:**
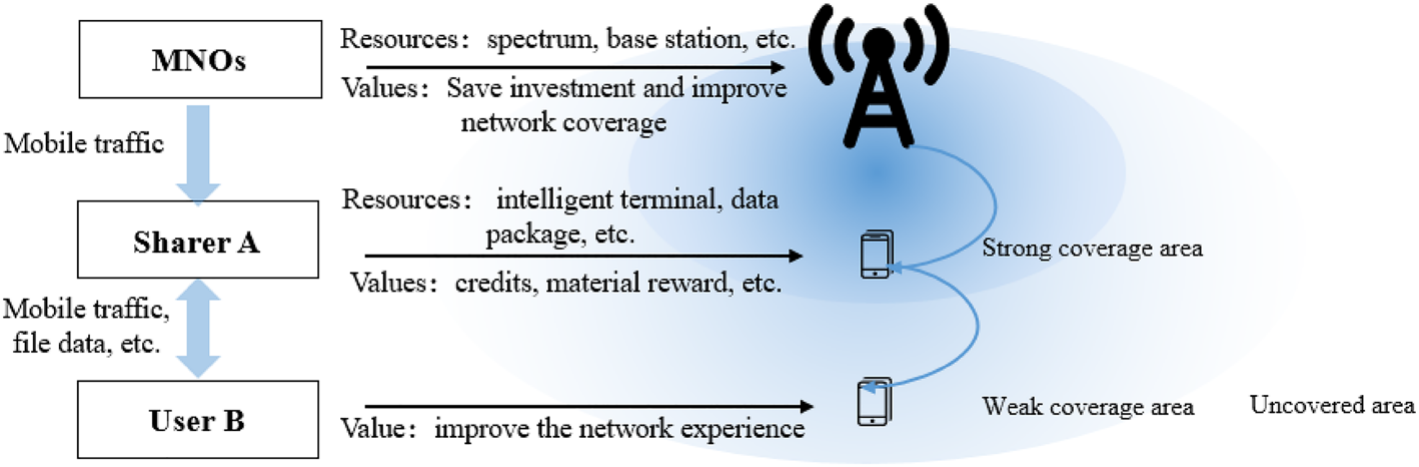
P-RAN value delivery process.

Based on cellular network and satellite network, MNO and sharers jointly build P-RAN to provide a 5G/6G enhanced network for users (as shown in Fig. 1). As can be seen from this scheme, the P-RAN network architecture mainly includes three: MNOs, users and sharers. These three interactive players have different value propositions, which will determine whether P-RAN can succeed in business. MNOs are concerned about whether P-RAN can get a higher return on investment (ROI). Users are concerned about whether P-RAN can offer them a better network experience. For the sharers, continuing to play the role of network repeater depends on whether there is an incentive system. Focusing on these three main issues, the value proposition of P-RAN is comprehensively analyzed, the evaluation system of value proposition is constructed, and the methodology that can be popularized is formed.

Value proposition design is based on generating and discussions about value creation^33^. Therefore, the content and method of the value proposition evaluation system in this paper mainly refer to the theoretical research results of predecessors.

As for MNOs’ value proposition, Kim and Mauborgne^34^ believe that the value obtained by enterprises comes from cost and price. Teece^35^ argued that the company’s value proposition creates financial returns for the company by developing, enabling and utilizing capabilities within the company. Emerson^24^ argued that the core essence of investment and return is the pursuit of an intrinsic value proposition composed of both. Since the corresponding products of P-RAN has not been listed, for the convenience of subsequent analysis, this paper will analyze the cost and benefit of P-RAN for MNOs from the perspective of return on investment finance.

For the customers’ value proposition, the primary indicator mainly refers to previous theoretical research and combines the consumer value theory put forwarded by Sheth et al.^36^, Kim and Mauborgne^34^, which stated that consumer value is composed of consumer utility and price. Thus, price and cost are the two main focuses of the primary indicators in this paper. Clarke III^37^ believes that mobile commerce value proposition is based on the following specific dimensions: ubiquity, convenience, localization and personalization. Berthon et al.^38^ identified five factors of employer attractiveness, namely interest value, social value, economic value, development value and application value. Rintamäki et al.^39^ develop a value proposition framework based on economic value, functional value, emotional value, symbolic value, or the combination of these value dimensions. Venkatesh et al.^40^ combined eight models to propose the Unified Theory of Acceptance and Use of Technology (UTAUT), including: performance expectations (perceived usefulness, competence, external motivation, relative advantage, outcome expectations), effort expectations (perceived ease of use, complexity, ease of use), social influences (subjective norm, image, social factors), convenient conditions (perceived behavioral control, facilitating conditions, compatibility), attitudes toward technology (attitude toward behavior, intrinsic motivation, affect toward use, affect). A comprehensive consideration of the MNO's value proposition and the customer's value proposition is helpful to judge the specific development direction of 6G products in the future, so this paper summarizes the primary indicators as shown in Table 1.

**Table 1:** Primary indicators and their explanation.

	Primary indicator	Explanation	References
MNO value proposition	Cost and benefit	MNOs’ investment and return	Emerson^24^ Teece^35^
Customer value proposition	Network experience	Network experience can be analogized to performance expectations, meaning that users can meet outcome expectations when using the network	Venkatesh et al.^40^ Berthon et al.^37^
	Connection	Connection function can be analogized to social influences, meaning that the difference in the customer's perception of connection with acquaintances or strangers	Kambil et al.^17^ Berthon et al.^38^ Venkatesh et al.^40^ Rintamäki et al.^39^
	Convenience	Convenience can be analogized to effort expectations, meaning that the ease of use, complexity of the customer in operating and using the network	Clarke III^37^ Venkatesh et al.^40^
	Compatibility	Compatibility can be analogized to convenient conditions, meaning that the degree of compatibility with network adaptations	Venkatesh et al.^40^ Berthon et al.^38^
	Added value	Added value can be analogized to the attitudes toward technology, meaning that the customers’ attitudes toward the network	Venkatesh et al.^40^ Rintamäki et al.^39^
	Special network usage scenarios	Considering the uniqueness of the Internet, user has different usage needs in different situations	Clarke III^37^
	Cost advantage Sharing revenue	Cost advantages and shared benefits can be analogized to the two most fundamental aspects of customer value, customer utility and cost	Sheth et al.^36^ Kambil et al.^17^ Berthon et al.^38^ Anderson et al.^41^ Rintamäki et al.^39^

Regarding the determination of secondary indicators, this paper believes that the project characteristics should be considered and discuss one case at a time. Therefore, it is determined by discussing brainstorming with experts.

In terms of evaluation methods, Anderson et al.^41^ put forwarded three methods for constructing the value evaluation system. Based on these methods, the research methodology of this paper is determined:

- Firstly, by listing all the product advantages, especially the utility that customers can get, evaluate the overall value of P-RAN.
- Secondly, by publicizing the advantages of this product compared with other products, highlight the utility that this product can bring to customers different from other products, judge the advantages of P-RAN and hot spots, broadband and mobile networks.
- Finally, by emphasizing the core pain points and resonance points of customers, provide suggestions for MNO to discover the potential value of P-RAN.

With the development of communication technology, new product has an overwhelming advantage over old products in performance index, which makes the performance index play a key role in network research and development (R&D), publicity and listing. In existing studies, most value research is based on QoE index. There is, however, limited research on the potential and unknown value proposition brought about by a network system with weak technical index. Exploring the interactive willingness and value proposition of each player in the network system for emerging networks is conducive to the construction and development of future networks. The value theory of Sheth et al.^36^ and Venkatesh et al.^40^ are only limited to publicizing the products that have already been listed. Although Anderson et al.^41^ provided an idea for the promotion of new products, P-RAN has certain particularity in research and development, listing, publicity and so on. As a product beneficial to the future, P-RAN needs to be realized by MNO, and it has certain policy and technical barriers. Therefore, it is of great significance to take P-RAN as an example for research. Furthermore, the analysis from the perspective of network interaction value is important to improve the technology-based evaluation methods in the construction of network systems.

## Construction of P-RAN value evaluation system

According to the above analysis, business innovation can be analyzed through the value proposition evaluation table. This paper uses semi-structured interviews with experts to conduct research and analysis. As 6G related research is relatively advanced, and there is no P-RAN or 6G product has been released yet, this paper will focus on interviews with experts in the field of wireless communications. In addition, in order to further explore business innovation and practice, this paper will also interview strategic value and operation experts. This paper invites 25 experts. These experts have combined their project experience and knowledge about P-RAN networks to make a comprehensive assessment about the evaluation metrics of the constructed MNO and customer value proposition based on relevant evaluation indicators and future development prospects. Due to the impact of the coronavirus, the main form of the interview is online conference. In order to ensure the objectivity of the interview, the interviewees and the interviewed experts are anonymous to each other, so as to minimize the possible influence of the interviewees' authority on the survey results. The duration of the interview ranges from 45 min to 1 h. During the interview, each expert was asked questions and scored according to the primary and secondary value evaluation index of the value proposition evaluation table. In order to avoid the error between the interviewer's rating and the actual opinions of the interviewed experts, the survey results were confirmed with the experts twice through email before they were finally identified as qualified questionnaires. In order to make the expert interview scientific and effective, and to make the survey results accurately reflect the value proposition of P-RAN, the interviewed experts were strictly screened and evaluated before the survey. The composition and qualifications of the interviewed experts include the following two points:

1. Experts can cover all fields of the industry: A total of 25 experts were interviewed, including 18 wireless network technology experts, 5 strategic value analysis experts and 2 MNO operation experts. The proportion of experts in various fields is basically consistent with the survey questions.
2. The expertise of the experts meets the requirements: All the experts have more than five years of work experience, and 9 of them have worked in the corresponding field for more than 10 years. All experts have a doctor's degree or senior engineer title or above.

This research used a collection of questionnaire forms to assess the importance of secondary indicators. The scores were scored on the Likert scale, in which a higher score means that indicator is more important. The value of the primary indicator is calculated by the arithmetic average of the second-level value evaluation index. Meanwhile, to ensure that experts can make clear judgments, the questionnaire in this paper is designed in such a way that experts can make comparative judgments between P-RAN and existing Wi-Fi broadband networks, 5G/6G mobile networks, and hotspots. By comparing and then ranking different network technology implementations of the same indicator, the importance of the indicator for P-RAN can be quickly judged. Finally, in this paper, considering that P-RAN network is the first-level entry method of cellular network, the overall performance of future 6G network will be greatly improved compared with the existing 5G network. Therefore, this paper distinguishes P-RAN networks into 5G and 6G, and discusses them separately.

### Analysis of MNO value proposition

According to the consumer value theory mentioned in the previous section, the value obtained by the firm is derived from the cost and price of the product. Since the ROI ratio of each MNO is relatively complex, it cannot be evaluated by a single numerical value. Therefore, this paper will mainly introduce the results of expert research from the ROI perspective. As the builder and promoter of P-RAN, analyzing the value of mobile network operators will be helpful to promote the construction and development of P-RAN. For MNO, the value of P-RAN mode is reflected in the enhancement of coverage and effective supplement of the current 5G network and even future 6G network; it is one of the green and low-carbon networking ways to save network investment. The value of P-RAN to MNO is analyzed from the perspective of cost and revenue^34^, that is the cost-performance evaluation (difference between resources, cost consumption, and direct and indirect income) is related to the three elements (key resources, cost structure and profit model) of the business model.

#### Evaluation of resources and cost input

According to expert interviews and financial indicators^24,35^, the cost input includes the following items: the resources and cost input of MNO include R&D cost, operation cost, network transformation cost, user incentive cost and other opportunity costs. According to the relationship between financial indicators, the formula is summarized as follows:

1 $$ \begin{aligned}   {{\text{C}}_{\text{cost}}} \; =  & \;{{\text{R}} \& {\text{D}} \, {\text{costs}}}\; + \;{{\text{Operating}}\; {\text{costs}}}\; + \;{{\text{Network}} \; {\text{transformation}} \; {\text{costs}}}\; + \;{{\text{Incentive}}\;{\text{cost}}} \\     &  + \;{{\text{Other}} \; {\text{opportunity}} \; {\text{costs}}} \\  \end{aligned}   $$

- R&D cost: It is applied to the development of P-RAN and platform to solve related technical problems, such as anti-interference, network security, privacy protection, and various difficulties in the construction of revenue sharing system.
- Operation cost: It is applied to business platform operation, network maintenance, marketing, security risk prevention, exception event handling, etc.
- Network transformation cost: It is used to upgrade the base station, spectrum and core network to make them compatible with P-RAN mode.
- User incentive cost: It is mainly used to encourage sharers to actively share P-RAN. For areas that need access to P-RAN, it can refer to take-away riders, Uber order distribution, etc. to build a dispatch mode, thus promoting sharers to take orders actively.
- Other opportunity costs: At present, MNO' cash cow products are whole-house Wi-Fi/ whole-house intelligence. These products have strong homogeneity with P-RAN in terms of functions and coverage, which may impact the existing business of MNO.

#### Evaluation of direct and indirect value income

Through the integration of P-RAN and mobile communication network, MNO can provide better network access experience for users, which has direct value and long-term potential value.

2 $$ {\text{V}}_{{{\text{value}}}} {\text{ = Direct value + Indirect value}} $$

Direct value includes:

- Improve the coverage of cellular network and save network investment: P-RAN is a low-cost networking mode; it can not only improve the coverage quality of cellular network, but also reduce the investment of network expansion and the need to build new base stations in indoor or outdoor weak coverage areas.
- Improve the utilization rate of cellular network: P-RAN alleviates the problem of insufficient network capacity or coverage in indoor or weak coverage areas by means of network capacity in strong coverage areas, and balances the utilization rate of network capacity in the whole network.
- Improve the utilization rate of user traffic: P-RAN mode allows users to share personal idle in-package traffic with other users, or through wholesale P-RAN special traffic packages from MNO, share them with other users in a paid way to improve traffic utilization rate.
- Improve user stickiness: P-RAN promotes users’ engagements through innovative incentive mode and expands users’ propaganda scope. Users will earn credits, physical rewards and other revenue from P-RAN sharing. These rewards will encourage users to continue to share, forming a virtuous cycle.

Indirect value includes:

- Expanding business opportunities: P-RAN will incubate new vertical industry applications, such as proximity services, point-to-point data transmission, emergency communication, etc.
- Commercial reserve of future technologies: P-RAN will explore a personal-centered, low-cost proximity networking model for future communication technologies such as 6G, and explore a low-cost, distributed, decentralized architecture for building a computing network platform.

The Benefit Analysis of MNO:

3 $$ {\text{V}}_{{{\text{revenue}}}} {\text{ = V}}_{{{\text{value}}}} - {\text{C}}_{{{\text{cost}}}} $$

At the initial stage of R&D and construction of P-RAN, MNO can introduce it into market at a lower cost through the intelligent terminal APP. The initial cost of the project is mainly focused on R&D and promotion, and the direct value may not fully cover the cost. However, network products have economies of scale. With the gradual improvement of cellular network quality in weak coverage areas, the number of customers will increase exponentially. With the increase of sharers scale and users scale, the potential value brought by the follow-up will continue to increase, and P-RAN will continue to bring positive benefits to enterprises.

### Analysis of user value proposition

Through the analysis of users’ value proposition, it will help MNO to recognize the differences of users’ value proposition, and formulate targeted publicity measures, so as to enhance the direct value and potential value.

The value proposition analysis of users and sharers is still done by expert research method. Considering that sharers also have the behavior of users, thus the usage behavior of sharers will not be analyzed separately.

#### Users

As the ultimate experiencer of the network, the users is the acquirer in the value chain and the value of network lies in its ability to meet the users' daily and entertainment requirements. Based on this premise, we define the primary and secondary value evaluation index of users from the aspects of usage expectation, functional value, social value, conditional value, convenience conditions, etc., as shown in the following Table 2:

**Table 2:** Definition of the user value proposition evaluation.

Primary value evaluation index	Secondary value evaluation index
Convenience	Convenience of operation Ask for otners’ help (for example, hotspots)
Network experience (security only)	QoE: network speed, stability, positioning accuracy, coverage area, safety Demand for SIM card: Whether a SIM card is required
Cost advantage	Working cost
Connection	Private domain connection Public domain connection
Special network usage scenarios	–
Compatibility	Adapter Terminal Type

From the convenience point of view, as a new type of network, P-RAN needs to realize the insensitivity in operation and use, so as to reduce the learning cost of users and achieve the purpose of rapid popularization. Analogous to benchmarking network, users need to enter the account password when using P-RAN for the first time, and then the system can connect automatically without any additional operations. At present, the operation difficulty of P-RAN is the same as that of Wi-Fi and hotspots. The future development direction of P-RAN can refer to the mobile network, and users can directly surf the internet without authentication.

From the perspective of network experience, specific index can be accurately compared and analyzed through QoE standards. The expert survey results are shown in the Table 3. P-RAN is comparable to or even superior to hotspots and Wi-Fi in terms of stability, coverage, positioning accuracy and security. Compared with mobile network, although P-RAN has advantages in the necessity of SIM card, it still has obvious deficiencies in network speed. This is because there is a problem of signal attenuation in the process of multi-hop transmission, and it is inevitable that the end user's network experience will decline. In the future, P-RAN should benchmark with mobile network and improve the network experience through various technical means.

**Table 3:** P-RAN, hotspot, broadband, mobile network (5G/6G) performance comparison.

Second-level value evaluation index	P-RAN 5G (Cell edge under mobile network coverage)^1^	R-RAN 6G Evolution direction (The access method is not only the cellular network, but can be accessed through broadband, WLAN, etc.)	Smartphone hotspots	Wi-Fi (exclude WLAN)	5G	6G
Network speed	Single-hop: < Cellular network;	Single-hop: < Cellular network;	< Cellular network;	–	–	–
Stability	Currently comparable to hotspots	Highest	Medium	High	High	High
Positioning accuracy	Currently comparable to hotspots	Highest	–	10 m	1 m	Indoor: 10 cm; Outdoor: 1 m
Coverage	Multi-hop: better than broadband Single-hop: comparable to hotspots	Become independent network coverage	10 m	50 m	Key areas are covered	Hot cover
Security	More secure than Wi-Fi	Highest	Medium	Medium	High	High
Demand for SIM card	No. The first login requires card verification	No	No	No	Yes	Yes
Price	local users: 0 Non-local users: < Wi-Fi	–	0	40 (ARPU)	87.35 (ARPU)	–

Description of "High-Medium–Low" grading standard: The grading standard of each indicator capability is determined from the perspective of whether there is a platform or means to guarantee QoE. In a comparison of several comparable technologies, normally the stronger the means of protection and the higher the perceived experience gained by the user, the higher the grading of the indicator capability.

Classification of stability: The classification of levels is based on whether there is a platform or means to control and guarantee the stability of network access and transmission rates within the coverage area. For example, compared with hotspots, the user's perception of Wi-Fi is higher rate and more stable within the same coverage distance. The network operation and maintenance of P-RAN, 4G and 5G are undertaken by MNOs, whose guarantee means are stronger and users perceive higher rate stability. In the future, 6G technical capabilities will be significantly enhanced, and the highest guarantee for stability is expected.

Classification of security: Technical, operational and policy factors are taken into consideration and the level is classified according to: whether the technology is secure in terms of control center, network connection method, transmission process, etc.; whether the MNO is qualified to operate; and whether the policy meets the policy compliance requirements.

In terms of costs, MNO does not charge the users of this network separately for using the P-RAN, but merges the usage fees into existing packages. For users in different MNO’ networks, it is also acceptable to get a better network experience at a lower price, which will become a new profit point for MNO. In the future, with the introduction of new technologies, MNO can explore more flexible charging methods.

On the connection function, P-RAN restores the geographical limitations of Wi-Fi and hotspots, and has the connection advantages comparable to that of mobile network, which can realize both public domain connection and private domain connection. The coverage area of hotspots and Wi-Fi is limited, which makes the user equipment unable to continuously connect to the network outside the signal range. The mobile network expands the geographical boundary between hotspots and wired networks, and user terminal can automatically connect to the network as long as it is within the coverage area of base station signals. P-RAN is as good as mobile network in coverage performance. Even without network coverage, P-RAN can help users realize point-to-point data transmission, which greatly expands the network usage scenario.

From the special network usage scenarios, P-RAN can transmit data in areas with poor signal or no signal, which greatly expands the coverage of existing mobile network. Sharing smartphone hotspot often happens among acquaintances. One party accesses the mobile network, and the others use the network for free, thus creating a debt of gratitude. Broadband-based Wi-Fi is suitable for transmitting large amounts of data in fixed places (such as remote work). In the 5G or 6G era, because the high cost of network construction, MNO can only achieve hotspot coverage basically. Overall, P-RAN, as a supplement to the mobile network, can meet the users' online needs anytime and anywhere.

From the perspective of compatibility, the first-level repeater of the P-RAN network, that is, the user equipment directly connected to the base station, must rely on the cellular network for data transmission. In addition, in terms of user terminal compatibility, MNO still need to overcome issues such as billing and connection. Among the existing wireless networks, Wi-Fi owns the most compatible terminals and application scenarios, which is the future development direction of P-RAN.

**Figure 2:**
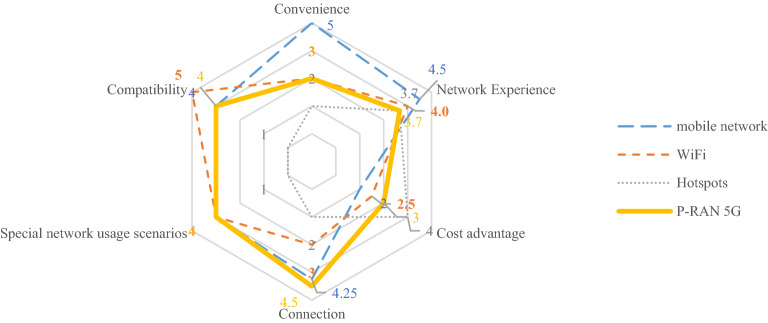
Results of value proposition evaluation of users.

Based on comprehensive analysis, we obtained the results of the primary indicators (corresponding Table 1) by arithmetic averaging the scoring results of the secondary indicators corresponding to the primary indicators, with the scoring values ranging from 1 to 5. The indicator with a higher score represents a greater differentiation advantage in performance and the higher value that can be realized. As shown in the Fig. 2 below:

The individual values and coverage area of the radar plot show the magnitude of each network's indicator value and overall value. The larger the coverage area, the higher the overall value of the network. The higher the score of an individual indicator, the higher the value of that indicator for that network. Overall, the commercial value of P-RAN is comparable to that of mobile network. In terms of special network usage scenarios and connection function, P-RAN has core advantages; P-RAN can still connect to the network automatically under the condition of weak coverage, and realize high-speed network transmission. Furthermore, user equipment can realize seamless handover among the mobile network, P-RAN and Wi-Fi. MNO can enhance the value of P-RAN by expanding proximity services such as encrypted file transmission in office scenarios.

The future P-RAN value proposition needs to be benchmarked against mobile networks and broadband networks. In terms of convenience, network experience, compatibility, etc., the value of P-RAN needs to realize the network experience consistent with the mobile network. In the future, MNO need to upgrade their technical solutions to reduce the problem of excessive signal attenuation in multi-hop connections, so as to improve the value perception of P-RAN. In the aspect of compatibility, MNO need to improve product compatibility and expand more available terminals. Finally, MNO will not charge users for using P-RAN services, but provide it to users as an additional benefit of mobile network package, which helps to create an image of cost-effective product value.

#### Sharers (repeater)

Sharers are an important node of P-RAN and play the role of value transmitters. P-RAN requires enough sharers to provide a great network experience for end users through multiple hops. Thus, increasing the perceived value of sharers is significant for MNO. The value of sharers needs to be considered from two aspects. On the one hand, the sharers themselves need certain network quality assurance when using the network, which is the same as the users’ value proposition. On the other hand, the sharers need a certain drive and guarantee to share the network, which can be cash, material rewards, or emotional rewards. Therefore, starts from the sharing behavior alone, this paper analyzes sharers, combined with users’ expectations, functional values, emotional values, convenience and other thinking directions, which eventually get the following Table 4:

**Table 4:** Definition of the sharer value proposition evaluation.

Primary value evaluation index	Secondary value evaluation index
Convenience	Login, operation, transaction, offline, account logout, option
Network experience (security only)	Safety is divided into commitment safety and health safety
Sharing revenue	Physical rewards, digital rewards Sharing revenue is divided into physical rewards received by the sharers and digital rewards, such as credits
Cost advantage	Online time, power consumption, terminal loss, and other service experience
Added value	Interest In addition to sharing revenue, users can get some interesting in the process of sharing through the marketing promotion of the company
Compatibility	Adapter terminal type

Convenience means that the easier and simpler it is to share the network, the more users will be attracted to participate in the sharing and construction of the network. Currently, P-RAN needs to register an account for sharing network. Access users can be selected according to whether a password is set, which is similar to Wi-Fi and hotspots (mobile network does not involve user sharing, so no comparative analysis is conducted). Wi-Fi can also select or restrict access users under special circumstances. The comparison with competing networks shows that in the future 6G era, P-RAN sharing needs to be simplifies in operation steps. Reducing operating costs and enabling “one-click sharing” can help it stand out from Wi-Fi and hotspots.

In terms of network experience, besides other indicators of QoE, P-RAN security can be comparable or even surpass Wi-Fi and hotspots. Driven by technology, in the future 6G era, P-RAN, as a mobile network operators-led network, has a security guarantee from MNO. Therefore, P-RAN can ensure that the sharers network nodes will not be attacked; traffic will not be stolen; privacy and security are protected; network radiation within the safe range and in accordance with national standards. Compared with Wi-Fi and hotspots without the support of MNO, P-RAN has higher security.

In terms of sharing revenue and cost, P-RAN has a clear competitive advantage in sharing revenue. Wi-Fi and hotspots are mostly free to share. Individual Wi-Fi can be charged by traffic through setting gateways. At the same time, hotspots sharing will significantly accelerate the power consumption and terminal loss of cell phones. There have been many studies on P-RAN network energy consumption. However, compared with competing network-products, there is still a terminal loss problem that can be perceived by sharers. At present, P-RAN can compensate for the loss of energy consumption through revenue and incentives. P-RAN sharers can share rewards such as credits, cash and physical objects obtained by network sharing. In the future, P-RAN combined with the research and development of new technologies will reduce terminal loss for sharers, which means reducing cost, is the direction of future technical efforts.

In terms of added value, since P-RAN is an enhanced network, MNO can increase sharers’ online sharing time through various incentives. Compared with Wi-Fi and hotspots, P-RAN network can adapt to more network application scenarios in the future, which has rich added value and potential value.

In terms of compatibility, P-RAN network does not have obvious advantages at present because it still requires the use of intelligent terminals and APPs to achieve. Competitive network Wi-Fi sharing requires the use of a router, and hotspots sharing requires opening the cellphone switch. If P-RAN can break through the limitation of sharing terminal in the future, it will help to realize the construction of comprehensive ubiquitous network.

**Figure 3:**
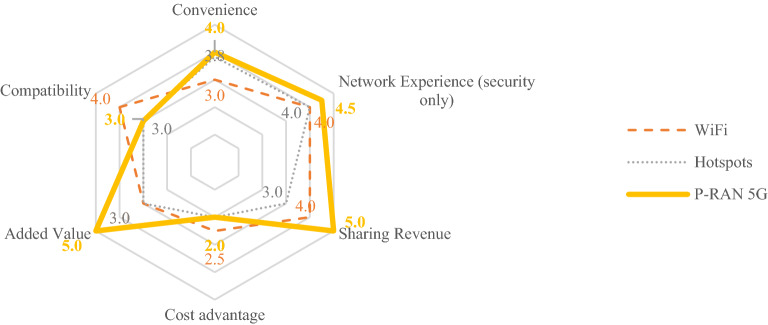
Results of value proposition evaluation of sharers.

Based on the above analysis, we compare the results as shown in the following Fig. 3:

In summary, the overall value proposition of P-RAN sharers at the present stage should surpass Wi-Fi and hotspots, showing outstanding performance in terms of added value and sharing revenue. In the early stage of P-RAN development, MNO highlight value differences and promoted user growth through sharing revenue incentives. MNO can also think from the perspective of potential application scenarios to improve sharers’ online time and promote added value growth.

In terms of convenience, network experience and compatibility, P-RAN is currently comparable to Wi-Fi and hotspots. However, MNO needs to provide security guarantee and technical support for P-RAN network sharing. MNO can use marketing propaganda to guide sharers to use idle terminals to share, which can reduce the impact of terminal loss and other problems. This will also improve the use rate of old terminals, reduce abandoned terminals, in line with the development trend of green environmental protection and low-carbon.

In the long run, terminal loss, energy consumption and diverse types of supporting devices in the sharing process are the key directions for future technological breakthroughs. MNO should ensure that the sharing operation is simple and break through the restrictions of terminal, APP, cost and other aspects under the condition of the sharer’s own online experience.

## Summary of P-RAN value proposition design

Combining with the previous analysis, it can be concluded that the value proposition of P-RAN’s landing implementation is as follows:

- P-RAN is a nationwide continuous indoor wireless connection scheme, which is comparable to or even superior to Wi-Fi (scattered indoor free broadband wireless connection scheme) in terms of convenience, network experience, compatibility and so on, and complement to mobile network. P-RAN users can form seamless connection switching among mobile network, P-RAN, Wi-Fi, and use them senselessly.
- P-RAN will focus on indoor areas with weak coverage or poor signal reception. At this stage, it can start from offices, buildings and other indoor areas. Users can register for the first time and subsequently connect directly to the internet no matter where or in any scenario.
- Compared with Wi-Fi and hotspots, P-RAN sharers have advantages in revenue. Sharing revenue helps MNO cultivate network sharers in the early stage. When enough sharers exist, the diffusion effect will be formed, which ultimately forms a “P-RAN + mobile network + Wi-Fi” network structure, expanding the coverage of the network and improving the ubiquitous network access capability.
- P-RAN has prominent advantages in connection function compared with Wi-Fi, hotspots and mobile networks. Combination of P-RAN’s connectivity and users’ mobile mode^42,43^ plays an important role in locating and delivering content, which will derive a number of near-domain service scenarios. For example, shopping malls can provide store advertisements and product price information based on users’ online information. During the epidemic, mobile information can be tracked to achieve precise prevention and control. Even in emergency rescue, P-RAN networks can be built quickly to achieve full coverage of network signals. P-RAN also has the characteristics of point-to-point off-network large data packet transmission, which enables data transmission and personnel check-in in office, school and other scenarios.
- With the iterative development of the network, P-RAN will become a 6G built-in function to share traffic. In the future, P-RAN can become an independent terminal network by expanding terminal types, providing a new development idea for MNO to seize market expansion.
- The realization of the value proposition of MNO, users and sharers is the closed-loop transmission process of value among the three, and is the key to whether P-RAN products can enter benign business operation, and is also the concrete embodiment of the value co-production approach in the future decentralization era. The realization of the value proposition of either party must depend on the other two parties. Users can continuously use P-RAN connectivity services, and the incentives and promotion efforts of MNO will affect the motivation of sharers to share the network and the growth of sharer volume. The volume of sharers and the quality of network sharing will influence the experience of users, and then affect the development scale of users. The growth rate of users and sharers will affect the willingness and motivation of MNO to expand the market, which in turn will affect the sharing revenue of sharers. It is because of the distributed network architecture of P-RAN that the relationship between nodes and nodes becomes equal. Compared with enterprise platform as the value co-production driver in the Internet era, every node in the future decentralized network is the value driver, which will realize the real equal value co-production.

## P-RAN 6G development recommendations

The construction of P-RAN conforms to the development trend of 5G and 6G mobile networks in the future. From the perspective of technology iteration process, the development of network to higher frequency is an inevitable direction. In the traditional cellular network architecture, high frequency and wide coverage has some disadvantages such as high cost, serious interference and large transmission loss. P-RAN can help to solve the problems of weak coverage caused by network signal passing through the wall, which will reduce the cost of network construction. Analyzing the interactive value proposition of P-RAN network provides a new idea for exploring other network systems to achieve value improvement. In addition, it also provides a thinking direction on operation and business innovation for future Web3.0 multi-party interactive distributed systems.

For MNO, P-RAN is a network product with potential to help drive innovation in networking and build a computing platform. P-RAN will not only reduce the cost of base stations but also save investment. P-RAN will become an effective complement to and increase coverage of 6G networks. Building a decentralized network helps to explore more business model scenarios for individuals and industries.

For network users, P-RAN has differentiated, long-term potential value. In the future, users will be able to seamlessly switch between “cellular network + P-RAN + Wi-Fi”, without perception. Thus, users can enjoy an internet experience that goes beyond Wi-Fi and tends to be mobile network. Besides, users can transfer large data from the network through P-RAN’s point-to-point features, which enables securely private information transmission in home, office and other places. Compared with Wi-Fi and hotspots, P-RAN sharers also have differentiated value. The establishment of sharing revenue mechanism promotes users to obtain digital assets relying on network resources.

Based on the previous theoretical basis, this paper designs a new evaluation standard for the construction of network development from the perspective of multi-party interaction value. It not only considers the relevant technical indicators, but also explores from the perspective of commercial landing. P-RAN is taken as an example for analysis and verification, leading to the development suggestions for MNO. This methodology helps MNO explore the future development direction of networking construction technology, clarify the advantages and disadvantages of P-RAN network in the future operation process, and plan relevant product operation activities.

## Contribution and future research perspectives

The significance and contribution of this paper at the theoretical level is through the design of P-RAN value evaluation model, extending the application of value co-production theory in the communication field, especially in mobile network construction. The existing literature focuses on value and business model related, mostly on the relationship between value and business model, or value discussion based on one stakeholder, but rarely seen a comprehensive stakeholder analysis and discussion. Through the design of 6G P-RAN value proposition, this paper demonstrates that value proposition is one of the important elements of business model. Meanwhile, this paper provides abundant theoretical support for the comprehensive consideration of enterprise value proposition and customer value proposition changes, and also provides theoretical reference for exploring the development direction of mobile network applications supported by high bandwidth, high speed and low latency network systems in the future 6G era.The analysis in this paper provides a reference method for the study of value co-production model in which each node is in an equal state. Meanwhile, this paper further enriches the connotation and application scenarios of value co-production theory, and makes innovations with practical aspects. Furthermore, this value evaluation method provides a systematic means of value evaluation for future judgments involving customer participation in network formation, which is of general significance.

The significance and contribution of this paper at the practical level is through the construction and analysis of the user and sharer value evaluation index system, providing a comparative assessment of P-RAN and other similar technologies. The advantages of P-RAN and the aspects that can still be improved in the future can be seen in the evaluation. In the future commercialization, these product advantages can be highlighted so that customers can quickly understand the product and form a good perception. In the meantime, the aspects of P-RAN that are not superior to other technologies in the evaluation provide the direction for future iterative development and optimization of the product.

Due to the limitation of time and effort, the research in this paper focuses on the construction of the P-RAN value evaluation model. However, certain value realization issues that affect the development of P-RAN are not within the scope of this paper, and these may serve as directions for future research. On the one hand, some technical and policy issues faced by P-RAN, such as how to obtain policy support for spectrum resources required by P-RAN, how to improve the protection of privacy and data security by P-RAN, how to judge weak coverage areas, and how to achieve optimal deployment of network resources, still need to be further explored and studied. On the other hand, further research can be conducted on some commercial issues before P-RAN enters the market in the future, such as how MNO can balance the relationship between P-RAN and existing intelligent networking products, and how to design the incentive mechanism for sharers to promote more sharers to join P-RAN. The gradual resolution of these issues in future research will be able to provide a series of valuable references and suggestions for the actual commercial operation of P-RAN.

## Data Availability

The data that support the findings of this study are available from China Telecom but restrictions apply to the availability of these data, which were used under license for the current study, and so are not publicly available. Data are however available from the authors upon reasonable request and with permission of China Telecom. If someone wants to request the data from this study, please contact corresponding author.
